# Transcriptome Analysis Reveals the Genes Involved in Oxidative Stress Responses of Scallop to PST-Producing Algae and a Candidate Biomarker for PST Monitoring

**DOI:** 10.3390/antiox12061150

**Published:** 2023-05-25

**Authors:** Xiangchao Zhang, Xiaogang Xun, Deting Meng, Moli Li, Lirong Chang, Jiaoxia Shi, Wei Ding, Yue Sun, Huizhen Wang, Zhenmin Bao, Xiaoli Hu

**Affiliations:** 1 MOE Key Laboratory of Marine Genetics and Breeding, College of Marine Life Sciences, Ocean University of China, Qingdao 266003, China; 2 Laboratory for Marine Fisheries Science and Food Production Processes, Qingdao National Laboratory for Marine Science and Technology, Qingdao 266237, China; 3 Key Laboratory of Tropical Aquatic Germplasm of Hainan Province, Sanya Oceanographic Institution, Ocean University of China, Sanya 572000, China

**Keywords:** paralytic shellfish toxins, scallop, digestive glands, oxidative stress, *C1QL4*, biomarker

## Abstract

Paralytic shellfish toxins (PST) could be accumulated in bivalves and cause safety problems. To protect public health, bivalves are examined for PST contamination before entering the market, usually by high-performance liquid chromatography (HPLC) or LC-tandem mass spectrometry (LC-MS/MS) in the lab, which needs PST standards not all available and is time-consuming for large sample sizes. To detect PST toxicity in bivalves rapidly and sensitively, a biomarker gene is highly demanded, but the related study is very limited. In this study, we fed a commercially important bivalve, *Patinopecten yessoensis*, with the PST-producing dinoflagellate *Alexandrium catenella*. After 1, 3, and 5 days of exposure, both PST concentrations and toxicity levels in the digestive gland continuously increased. Transcriptome analysis revealed that the differentially expressed genes were significantly enriched in oxidation-reduction process, which included the cytochrome P450 genes (*CYP*s), type I iodothyronine deiodinase (*IOD1*s), peroxidasin (*PXDN*), and acyl-Coenzyme A oxidase 1 (*ACOX1*) at day 1 and a superoxide dismutase (*SOD*) at day 5, highlighting the crucial roles of these genes in response to oxidative stress induced by PST. Among the 33 continuously upregulated genes, five showed a significant correlation between gene expression and PST concentration, with the highest correlation present in *PyC1QL4-1*, the gene encoding Complement C1Q-like protein 4, C1QL4. In addition, the correlation between *PyC1QL4-1* expression and PST toxicity was also the highest. Further analysis in another aquaculture scallop (*Chlamys farreri*) indicated that the expression of *CfC1QL4-1*, the homolog of *PyC1QL4-1*, also exhibited significant correlations with both PST toxicity and concentration. Our results reveal the gene expression responses of scallop digestive glands to PST-producing algae and indicate that the *C1QL4-1* gene might be a potential biomarker for PST monitoring in scallops, which may provide a convenient way for the early warning and sensitive detection of PST contamination in the bivalves.

## 1. Introduction

Paralytic shellfish toxins (PST), including saxitoxin (STX) and its over 50 analogs, are among the most toxic and widely distributed marine biotoxins in the world [1,2]. They are mainly produced by toxic dinoflagellates, especially during harmful algal blooms. As filter feeders, bivalves could accumulate PST through ingesting PST-producing algae [3,4]. Consumption of bivalves contaminated with PST (more than 3000 mouse units (MU)) may be life-threatening for humans and other animals [5]. In addition to their direct impacts on human health, PST also have a serious negative impact on shellfish aquaculture [6]. To ensure the security and quality of farmed bivalves, PST concentration and toxicity in bivalves are measured before bivalves enter the market [7]. Many countries set the regulatory limit of PST at 80 µg STXeq (100 g)^−1^, which is specified by the World Health Organization (WHO) and the Food and Agriculture Organization of the United Nations (FAO) [8]. To better protect consumers, some countries raised tighter vigilance standards, such as 60 µg STXeq (100 g)^−1^ in the Philippines [9] and 40 µg STXeq (100 g)^−1^ in Ireland and the Netherlands [10].

By far, the methods used for PST detection in bivalves mainly include mouse bioassay (MBA) [11], enzyme-linked immunosorbent assay (ELISA) [12], high-performance liquid chromatography (HPLC), and liquid chromatography-tandem mass spectrometry (LC-MS/MS) [13]. However, the sensitivity of the MBA is relatively low, and the assay faces ethical concerns because of animal distress [14]. ELISA is an antibody-based immunoassay, which is fast and easy to operate, but these antibody-based kits require an animal host for their production, and it is not easy to obtain antibodies against all PST homologues [5]. Compared with ELISA, HPLC and LC-MS/MS have improved the separation of the PST [15], and LC-MS/MS has been extensively used in PST examination for its superior specificity and sensitivity [16]. However, both HPLC and LC-MS/MS analyses require pure PST standards that are not commercially available for all the saxitoxin derivatives. Meanwhile, chromatographically resolving metabolites from complex biological samples is usually time-consuming [17], and the number of samples tested at one time is limited. The development of PST markers is essential for rapid and convenient PST monitoring.

Bivalves are one of the major sources of human dietary animal protein [18,19]. More than 70% of marine aquaculture production comes from shellfish farming in China, most of which are bivalves [20]. A rapid and sensitive PST examination is highly demanded for bivalve culture and marketing regulation. Compared with LC-MS, biomarkers are more rapid, highly sensitive, and less expensive. Analysis of gene expression profiles has made it possible to identify genes suitable for biomarker candidates. However, there was no systemic screening for the biomarker genes for PST monitoring in bivalves.

Recent studies showed that the digestive gland accumulated much more PST than other tissues in bivalves, and PST concentrations and toxicity levels in the digestive gland exhibited linear correlations with the whole soft-tissues. Thus, the digestive gland is a suitable organ for rapid and sensitive PST monitoring in bivalve [21] and for studying the response of bivalve to PST-producing algae [22]. Moreover, the accumulation of PST may induce oxidative stress, which results in an imbalance between the production of reactive oxygen species (ROS) and the antioxidant system [23,24,25]. However, the response to oxidative stress caused by PST in bivalves is mainly focused on a specific gene or a gene family [26,27,28], and the systematic analysis of genes involved in the oxidative stress response of scallops to PST-producing algae is still limited.

In this study, we found that the significantly upregulated genes in the digestive gland of *Patinopecten yessoensis* exposed to PST-producing algae were mainly enriched in oxidation-reduction, metabolism, and transport processes after 1, 3, and 5 days of exposure, respectively. Several genes involved in oxidative stress caused by PST-producing algae exposure were identified. Moreover, through transcriptomic data screening, we found that 33 genes were continuously upregulated in the digestive gland of massively cultured scallop, *P. yessoensis*, during exposure to PST-producing dinoflagellates, and the expression of *PyC1QL4-1* showed the highest correlation coefficient with both PST concentration and toxicity. Further analysis indicated that the expression of *CfC1QL4-1*, the *PyC1QL4-1* homolog, in another commercially important scallop, *Chlamys farreri,* also exhibited significant correlations with both PST concentration and toxicity. Our study suggests that *C1QL4-1* may be a potential biomarker gene for PST monitoring in scallops, and transcriptomic data represent useful resources for biomarker gene searching for PST monitoring in bivalves.

## 2. Materials and Methods

### 2.1. Culturing of PST-Producing Alexandrium catenella

We maintained PST-producing *A. catenella* in 5 L flasks with f/2-Si culture medium, which was prepared with autoclaved and filtered natural seawater (pH 7.9 ± 0.1, salinity 30 ± 1). The *A. catenella* was preserved at 23 ± 1 °C with cool white illumination (90 μmol photon m^−2^s^−1^). Except for these, a 14:10 (light:dark) cycle was needed. A fraction of the *A. catenella* culture was collected every day for cell counting using the Countstar^®^ BioTech Automated Cell Counter and PST analysis.

### 2.2. Exposure of P. yessoensis to PST-Producing A. catenella

To set up toxin accumulation trials, we first acclimated *P. yessoensis* in an aquarium tank (300 mm × 300 mm × 500 mm) with static aerated seawater at 12–13 °C for three weeks by feeding no-toxic *Isochrysis galbana*. Then each scallop was fed with *A. catenella* once a day with a final cell density of 2500 cells mL^−1^. Three scallops were randomly collected at day 0 (control), day 1, day 3, and day 5, respectively, after exposure. For each sampled scallop, the digestive gland was separated from the scallop and then weighed and stored at −80 °C for the subsequent transcriptome and PST analyses.

### 2.3. Measurement of the PST Concentration and Toxicity

The digestive gland was freeze-dried for 24 h and then manually grounded with a stainless medicine spoon. 2 mL 0.1% formic acid was added to 1 g of digestive gland powder and mixed thoroughly. After 48 h, the mixture was centrifuged at 12,000× *g* for 10 min at 4 °C, and then the supernatant was collected and cleaned over an Oasis^®^ HLB Extraction Cartridge, which has been treated with 6 mL methanol and 6 mL water. The filter liquor was collected and mixed with an equal amount of acetonitrile; then the mixture was centrifuged at 12,000× *g* for 10 min at 4 °C. Finally, the supernatant was collected and filtered through a 0.22 µm membrane. The supernatant was decanted into a 2 mL brown vial for the following LC-MS analysis.

PST in *A. catenella* and digestive glands were determined by using a QTRAP 4500 mass spectrometer (AB SCIEX, Framingham, MA, USA), equipped with an ESI source and an ExionLC AC HPLC. The chromatographic separation was performed on a TSK-gel Amide-80 column (3.0 μm, 2.0 mm × 250 mm) at 30 °C, eluted at 0.25 mL min^−1^. Mobile phase A was water and B was 95% acetonitrile aqueous solution. Both A and B contain 2 mmolL^−1^ ammonium formate and 7.2 mmolL^−1^ formic acid (pH = 2.5). The elution time program followed the protocol described by Meng et al. [21].

The toxicity and PST concentrations were calculated according to the national standard GB 5009.213-2016 (Determination of Paralytic Shellfish Poison in Shellfish). The overall PST concentration (*C*) was obtained to determine the degree of PST accumulation per unit of tissue mass (ng/g).

$$C=\frac{\sum_{i=1}^{n}ci\times ri}{w}\times V_{\mathrm{extract}}\;\times \;1000$$

where *c_i_* represents the concentration (μmol L^−1^) of PST analog *i* in the extracted solution, *w* was the weight of the digestive gland (g), *V*_extract_ was the total volume (L) of the extracted solution, and *r_i_* was the molecular weight of PST analog *i*.

Then the overall toxicity of PST in the digestive gland was calculated (µg STXeq (100 g) ^−1^).

$$\mathrm{Toxicity}=\frac{\sum_{i=1}^{n}Ci\times Fi}{w}\;\times \;372.2\;\times \;100$$

where *Ci* was toxin content (μmol) of PST analog *i* in a specific tissue, *w* was the weight (g) of a specific tissue, *Fi* was the relative toxicity of PST concentration *i* to saxitoxin, and 372.2 was molecular weight of saxitoxin dihydrochloride (Table 1).

### 2.4. Transcriptome Analysis of the Digestive Gland in P. yessoensis Exposed to PST-Producing A. catenella

Total RNA was extracted from digestive glands using the guanidinium isothiocyanate method [29] and then digested with DNase I (TaKaRa, Shiga, Japan) to remove residual DNA. At each time point, three individuals with high-quality RNA were selected to construct the library. The transcriptome library of the scallop digestive gland was constructed using the NEB Next mRNA Library Prep Kit. The concentration of the constructed RNA-seq library was determined by a Qubit RNA Assay Kit (Invitrogen, Carlsbad, CA, USA). The prepared libraries were subjected to paired-end 125 bp (PE125) sequencing on the Illumina HiSeq 2500 platform.

The high-quality reads of each sample were aligned to the genome of *P. yessoensis* (GeneBank: GCA_002113885.2) [30]. The counts of reads mapped to each gene were obtained using HTSeq [31]. Reads Per Kilobase per Million mapped (RPKM) values based on read counts and transcript lengths were used to evaluate the expression level of each gene. The gene expression profile was compared between the digestive glands sampled at each of the exposed time points and the control group. The differentially expressed genes (DEGs) were obtained by the Bioconductor package edgeR (v3.6.1) in R language, using the threshold log_2_|FC| ≥ 2 and *p* < 0. 05. With the GO (gene ontology) [30], the enriched GO terms (GO level = 4) in the DEGs were analyzed by the Enrich Pipeline [32].

### 2.5. Screening and Characterization of the Candidate Biomarker Gene in P. yessoensis for PST Monitoring

For the DEGs, cor.text in R was used to calculate the correlation between gene expression and PST concentration and between gene expression and PST toxicity [33]. Correlations were considered significant if *p* < 0.05 to select candidate biomarker genes for PST monitoring. The candidate genes were annotated by the Swissport database [30]. The conserved domains were predicted by the simple modular architecture research tool (SMART) [34] and the protein structure map was drawn by IBS (Illustrator of biological sequences) [35]. The secondary structure of the protein was predicted and plotted by Geneious4.8.3 software [36]. The expression patterns of candidate genes in nine adult tissues (mantle, foot, gill, kidney, female gonad, male gonad, smooth muscle, striated muscle, and digestive gland) were analyzed using the transcriptomic data of *P. yessoensis* [30].

### 2.6. Verification of the Candidate Biomarker Gene in C. farreri

For the candidate biomarker gene identified in *P. yessoensis*, its homologous in the genome of *C. farreri* was further analyzed for the correlation between the gene expression and PST concentration and between the gene expression and PST toxicity, using cor.text in R. The correlations were considered significant if *p* < 0.05. The expression patterns of the gene were analyzed in nine adult tissues (mantle, foot, gill, kidney, female gonad, male gonad, smooth muscle, striated muscle, and digestive gland) using the transcriptomic data of *C. farreri* [22].

## 3. Results

### 3.1. PST Composition and DEGs in the Digestive Gland of P. yessoensis after A. catenella Exposure

**Figure 1:**
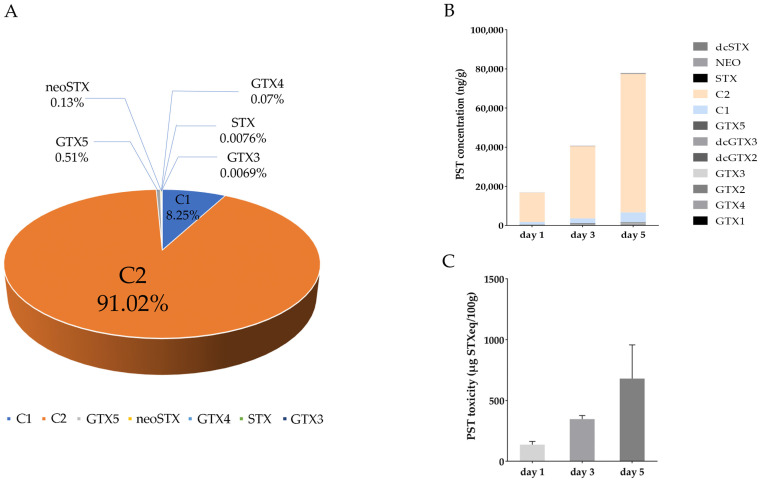
The proportion of PST analogs in *A. catenella* (**A**). The concentration (**B**) and toxicity (**C**) of PST in the digestive gland of *P. yessoensis* after exposure to *A. catenella*.

LC-MS analysis indicated that over 99% of the PST produced in the toxic *A. catenella* were N-sulfocarbamoyl-gonyautoxin-3 (C2) and -2 (C1), with the concentration of C2 being 10 times higher than C1 (C1: 0.07 μmol, C2: 0.82 μmol) (Figure 1A). In the digestive gland of *P. yessoensis* exposed to *A. catenella*, C2 was also the dominant PST, and PST concentration and toxicity were continuously increased as exposure time went on (Figure 1B,C). After 1, 3, and 5 days of exposure, PST concentration was 1.69 × 10^4^ ng/g, 4.07 × 10^4^ ng/g, and 7.79 × 10^4^ ng/g, respectively, and PST toxicity was 136.33 µg STXeq/100 g, 346.80 µg STXeq/100 g and 679.72 µg STXeq/100 g, respectively.

**Figure 2:**
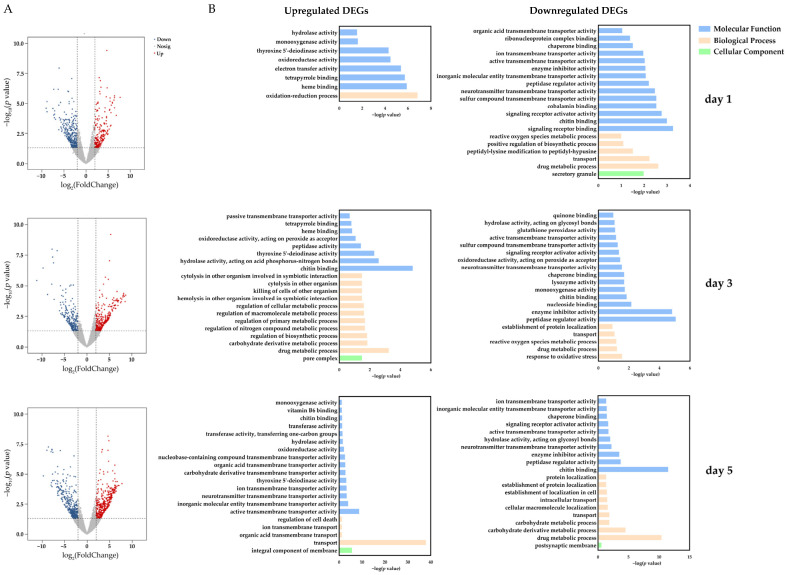
Volcano plots (**A**) and GO enrichment results of DEGs (**B**) in the digestive gland of *P. yessoensis* exposed to *A. catenella*.

To identify the genes response to the exposure of PST-producing algae, we compared the transcriptome data of the digestive gland between the samples at each of the time points (days 1, 3, and 5) and the control (day 0). A total of 532, 546, and 750 DEGs (log_2_|FC| ≥ 2, *p* < 0.05) were identified at day 1, day 3, and day 5, respectively, compared to day 0 (Figure 2A). Among the DEGs, 201, 311, and 377 were upregulated, and 331, 235, and 373 were down-regulated, at day 1, day 3, and day 5, respectively (Figure 2A).

**Figure 3:**
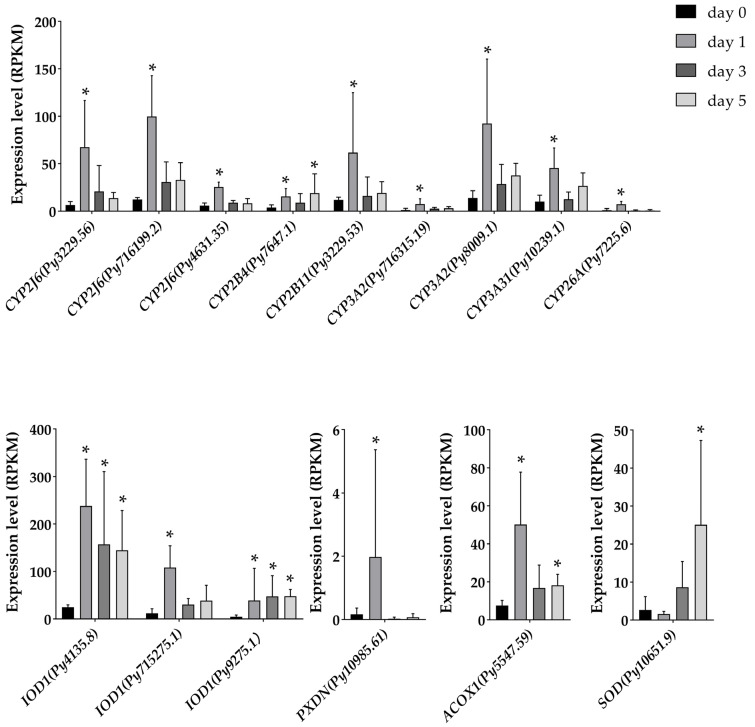
The expression of *CYP*s, *IOD1*s, *PXDN*, *ACOX1*, and *SOD* in the digestive glands of *P. yessoensis* during the exposure to *A. catenella* (* indicates *p* < 0.05 and log_2_|FC| ≥ 2).

We further explored the function of the upregulated and downregulated DEGs through GO enrichment analysis (Figure 2B). The results showed that at day 1, 201 upregulated DEGs were enriched into eight GO terms, and the most significant term was the oxidation-reduction process, followed by the term of heme binding. The DEGs involved in the oxidation-reduction process included the members of the cytochrome P450 (*CYP*s) family, type I iodothyronine deiodinase (*IOD1*s), peroxidasin (*PXDN*), as well as acyl-Coenzyme A oxidase 1 (*ACOX1*). A *CYP2J6* gene (*Py716199.2*) was the most significantly upregulated gene (*p* = 7.56E-8) among all the 201 upregulated DEGs, which exhibited the highest expression at day 1 compared with other time points. Similar expression patterns were also observed in other *CYP* members, including two other *CYP2J6*s, two *CYP2B*s, two *CYP3A2*s, one *CYP3A31*, and one *CYP26A1* (Figure 3). Except for *CYP* genes, three *IOD1* genes, which converted T4 (tetraiodothyronine) to T3 (triiodothyronine) [37], were also enriched in the oxidation-reduction process, and two of them showed significant upregulation at day 1, day 3, and day 5 (Figure 3). Besides, a peroxidase family member, PXDN, which can directly or indirectly oxidize numerous organic and inorganic substrates by consuming hydrogen peroxide (H_2_O_2_), was found to be upregulated at day 1. Another enzyme, ACOX1, which functions in maintaining redox and lipid homeostasis, was also upregulated at day 1 and day 5 (Figure 3). Meanwhile, the 331 downregulated DEGs were enriched into 34 GO terms, and the most significant GO term was the signaling receptor binding. Among the 331 downregulated DEGs, the most significantly downregulated gene was the dual-oxygenase maturation factor (*DUOXA1*), an activator of dual-oxygenase 1 (*DUOX1*).

At day 3, the 311 upregulated DEGs were enriched into 39 GO terms. The most significant GO term was chitin binding, followed by the term of drug metabolic process. Moreover, there were 15 GO terms related to the metabolic process, which indicated that the activation of the metabolic process was the main response of the scallop digestive gland after 3 days of PST-producing algae exposure. Among the upregulated DEGs, indoleamine 2,3-dioxygenase 1 (*IDO1*) was the most significantly upregulated gene, followed by *IOD1*. Meanwhile, the 235 downregulated DEGs were enriched into 46 GO terms, and the most significant term was peptidase regulator activity, followed by the term of enzyme inhibitor activity. However, among the downregulated DEGs, the top 10 significantly downregulated genes were uncharacterized, followed by the 1,5-anhydro-D-fructose reductase (*Akr1e2*) gene.

At day 5, the 377 upregulated DEGs were enriched into 46 GO terms and were mainly involved in transport processes, including 26 solute carriers (*SLC*s). Among the upregulated DEGs at day 5, *IOD1* was the most significantly upregulated gene. Except those, an antioxidant gene, superoxide dismutase (*SOD*), was also significantly upregulated at day 5 (Figure 3), which plays an important role in attenuating oxidative damage [38]. As for the 373 downregulated DEGs, 56 GO terms were enriched, and the most significant term was chitin binding, followed by the term of drug metabolic process, and collagen alpha-6 (*COL6A6*) was the most significantly downregulated gene.

### 3.2. Screening and Characterization of the Biomarker Gene for PST Monitoring in P. yessoensis

**Figure 4:**
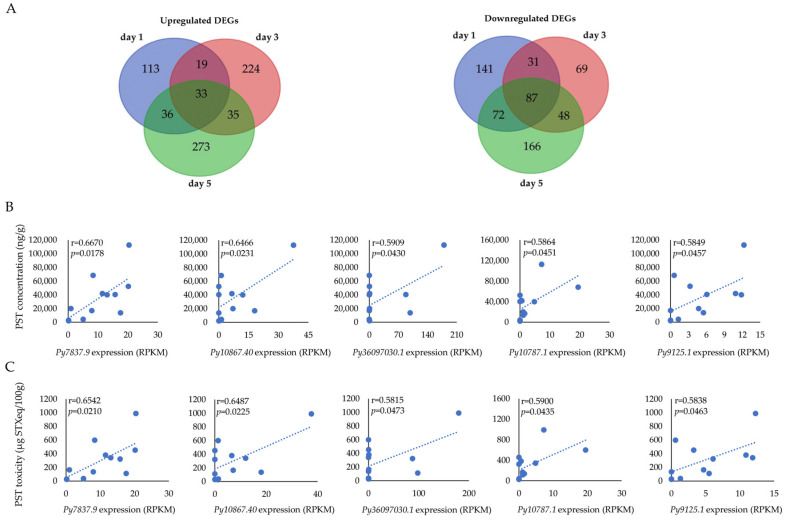
(**A**) Venn diagrams of significantly upregulated and downregulated DEGs in the digestive gland of *P. yessoensis* after exposure to *A. catenella*; (**B**) The correlation between the expression of continuously upregulated DEGs and PST concentration; (**C**) The correlation between the expression of continuously upregulated DEGs and PST toxicity.

Among the DEGs, a total of 33 genes were continuously upregulated and 87 genes were continuously downregulated during the whole exposure stage (Figure 4A). As for the 33 continuously upregulated genes, only 18 genes were annotated by the Swissport database. To identify the potential biomarker genes for PST monitoring, we analyzed the correlations between the expression levels of the continuously upregulated genes and PST concentration and toxicity in the digestive gland (Figure 4B; Tables S1 and S2). Five continuously upregulated genes showed significant correlations between gene expression and PST concentration, with the correlation coefficients being r = 0.6670, 0.6466, 0.5909, 0.5864, and 0.5849 for the genes *Py7837.9*, *Py10867.40*, *Py36097030.1*, *Py10787.1*, and *Py9125.1*, respectively. The gene *Py7837.9*, which showed the highest correlation coefficient, was annotated as Complement C1Q-like protein 4 (named *PyC1QL4-1*). Moreover, the expression levels of these five genes were also significantly correlated with PST toxicity, with correlation coefficients of r = 0.6542, 0.6487, 0.5815, 0.5900, and 0.5838 for the genes *Py7837.9*, *Py10867.40*, *Py36097030.1*, *Py10787.1*, and *Py9125.1*, respectively. The highest correlation coefficient was found in *PyC1QL4-1* again (Figure 4C).

**Figure 5:**
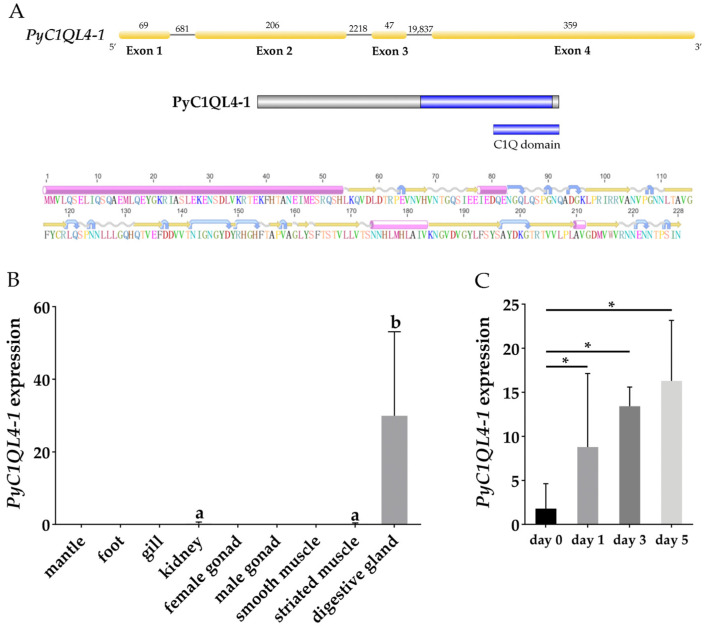
(**A**) The structure of *PyC1QL4-1* gene and PyC1QL4-1 protein. Yellow rectangles represent exons, gray lines represent introns, and numbers represent the length of introns and exons. The pink cylinders, yellow arrows, blue arrows, and wavy lines stand for α-helices, β-strands, turns and coils, respectively; (**B**) The expression profiles of *PyC1QL4-1* in adult *P. yessoensis* tissues. Values with different superscripts indicate statistical significance (*p* < 0.05); (**C**) The expression of *PyC1QL4-1* in the digestive glands of *P. yessoensis* exposed to *A. catenella* (* indicates *p* < 0.05 and log_2_|FC| ≥ 2).

The sequence of *PyC1QL4-1* was obtained from the genome data of *P. yessoensis* [30], which was 23,417 bp in length with four exons encoding 228 amino acids. A conserved C1Q domain [39] between the 124th and the 223th amino acids at the N-terminal was found. The secondary structure showed that the protein contained four α-helices, including a longer α-helix structure at the C-terminal (Figure 5A). The typical C1Q domain can form a jam roll shape composed of 10 β-strands and eight conserved amino acid residues that exist in the center of the C1Q domain [40]. Expression analysis indicated that, in the adult tissues of *P. yessoensis*, *PyC1QL4-1* was specifically expressed in the digestive gland (Figure 5B), which proved to be the most important organ for PST absorption in bivalves [21]. The specific and continuous upregulation of *PyC1QL4-1* in the digestive glands suggests that *PyC1QL4-1* might play an important role in PST responses in these PST-enriched tissues (Figure 5C).

### 3.3. Correlation of PyC1QL4-1 Homologs Expression and PST Accumulation in C. farreri

**Figure 6:**
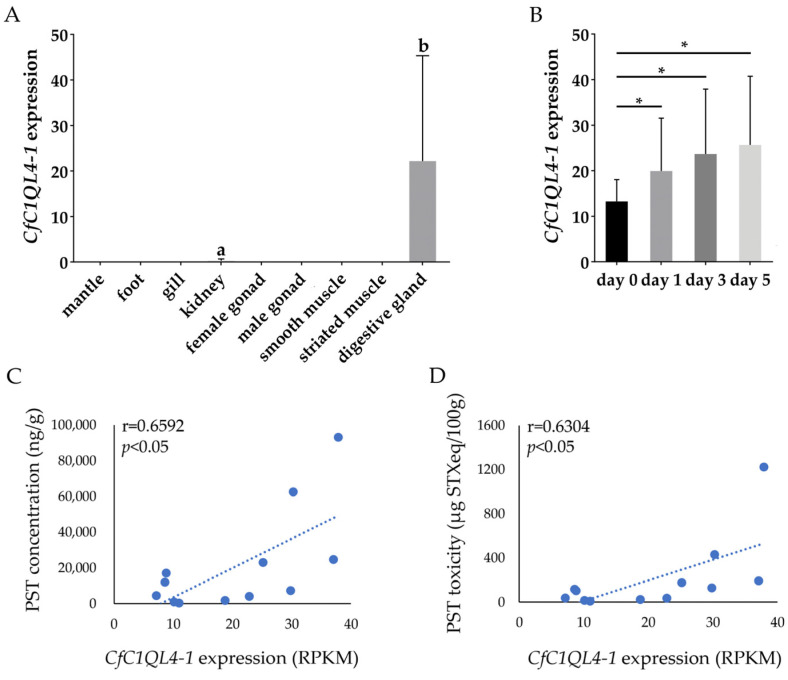
(**A**) The expression pattern of *CfC1QL4-1* in the adult tissues of *C. farreri*. Values with different superscripts indicate statistical significance (*p* < 0.05); (**B**) *CfC1QL4-1* expression in the digestive glands of *C. farreri* exposed to *A. catenella* (* indicates *p* < 0. 05); (**C**) The correlation between the *CfC1QL4-1* expression and PST concentration in the digestive gland of *C. farreri*; (**D**) The correlation between *CfC1QL4-1* expression and PST toxicity in the digestive gland of *C. farreri*.

We further screened the homologous gene of *PyC1QL4-1* in another commercially important scallop, *C. farreri*, and then analyzed the correlation between their expression and PST concentration in the digestive gland using the transcriptomic data of *C. farreri* after *A. catenella* exposure (data unpublished). The results showed that among the 56 *C1QL4* genes in the *C. farreri* genome, one gene (named *CfC1QL4-1*) showed the highest sequence similarity with *PyC1QL4-1* (identity percentage = 74.09%, *e*-value= 3.96 × 10^−115^) and it was also expressed specifically in the digestive gland (Figure 6A), and it was continuously upregulated after *A. catenella* exposure (Figure 6B). Moreover, the expression level of *CfC1QL4-1* was significantly correlated with both PST concentration (r = 0.6592, *p* < 0.05) and toxicity (r = 0.6304, *p* < 0.05) in the digestive gland (Figure 6C,D). Therefore, the gene *C1QL4-1* might be a candidate biomarker to assess PST contamination in scallops.

## 4. Discussion

In our previous studies, the digestive gland was found to be the major center for PST accumulation in scallops [22] and also suitable for sensitive PST monitoring in bivalves [21]. In this study, we analyzed the transcriptomic responses of the digestive gland in *P. yessoensis* exposed to PST-producing algae. In addition to revealing the oxidative stress response caused by the toxic algae, a candidate biomarker gene was found for PST monitoring in scallops.

After *P. yessoensis* was exposed to *A. catenella*, the upregulated DEGs at day 1 were most significantly enriched in the oxidation-reduction process. We analyzed the DEGs in this term and found that most of them are *CYP* family members, including a *CYP2J6* gene with the most significant difference among all upregulated DEGs at day 1. In previous studies, CYP enzymes played key roles in many crucial biological processes, including the oxidative transformation of xenobiotics and the metabolism of endogenous substrates, and they were regarded as markers of oxidative stress [41]. In this study, several *CYP* family members showed significant upregulation at day 1, indicating PST act as xenobiotics and pose oxidative stress during the exposure of scallops to *A. catenella*. We also found two upregulated enzyme proteins, PXDN and ACOX1, were involved in this term. PXDN belongs to a family of heme-containing peroxidases that catalyze the oxidation of various substrates, mainly utilizing the ROS, H_2_O_2_, in the formation of hypohalous acids, and are generally thought to be anti-oxidative enzymes [42]. It has been shown PST could induce oxidative stress in bivalve species through the overproduction of ROS during exposure [23,24]. The upregulation of *PXDN* indicates that this gene might be involved in protecting the scallops from the ROS damage caused by *A. catenella* exposure. ACOX1 is the first rate-limiting enzyme of the peroxisomal β-oxidation system [43]. The expression level of *ACOX1* was enhanced, suggesting that peroxisomal β-oxidation increased, which might represent another response mechanism for dealing with oxidative stress. Otherwise, the most significantly downregulated DEG was *DUOXA1,* which plays a positive role in the generation of ROS [44]. The downregulation of *DUOXA1* indicates that the generation of ROS may be inhibited during exposure to PST-producing algae. At day 3, the most significantly upregulated DEG was *IDO1*, which is characterized as a rate-limiting metabolic enzyme that converts L-tryptophan into downstream kynurenines, thus stimulating the kynurenine pathway that inhibits the proliferation of immune cells [45]. At day 5, the most significantly upregulated DEG was *IOD1*, which is a selenoenzyme that modulates thyroid hormone (TH) action by regulating the availability of T3 [46]. Meanwhile, TH is required for normal development as well as regulating metabolism in animals [47]. The upregulation of *IOD1* would accelerate the metabolic processes of *P. yessoensis* after exposure to *A. catenella*. Moreover, the significantly upregulated DEGs were mainly involved in transport processes, including 26 solute carriers (*SLC*s), which primarily mediate the absorption of small molecules into cells [48]. The processes of transport were significantly enriched, implying their possible involvement in PST transport or absorption. As for *SOD*, it is an antioxidant gene, and the role of this enzyme in modulating the cellular toxicity of superoxide has been well established [49]. The expression of *SOD* was significantly upregulated, which implied that the antioxidant process is a crucial response after exposure to *A. catenella.* Moreover, the most significantly downregulated DEG was *COL6A6.* Previous studies have shown that *COL6A6* could maintain cell structure integrity and suppress the growth of cells [50]. The *COL6A6* was significantly downregulated in *P. yessoensis* after exposure to *A. catenella*, which indicated that the PST might play a negative role in growth regulation of cells. In summary, the genes involved in the oxidation-reduction process play crucial roles in response to the oxidative stress induced by PST, and PST may have a negative impact on immune function and growth regulation in scallops.

To protect human health from the harm caused by consuming bivalves, contaminated with PST, PST detection is required during bivalve trading. By far, the main detection methods of PST mainly include MBA, ELISA, HPLC, and LC-MS/MS [11,12,13]. The MBA for the detection of marine biotoxins has been in use for 40 years; however, the mouse bioassay was considered to be replaced by other methods because of ethical and technical problems [51]. As for ELISA, it was employed to perform an initial screen on urine specimens for direct confirmatory testing, but it has the limitations of immunoassay testing [52]. HPLC and LC-MS/MS seem to be suitable for the detection of toxins, with a lower LLOQ (lowest limit of quantification) compared to ELISA in the present study [53]. However, the poor availability of standards, the lack of standards for all analogs, and the sample limit at one time may be the bottlenecks of HPLC and LC-MS/MS [54]. Compared with these methods, using molecular markers, especially biomarker genes, in toxin detection is a rapid, sensitive, and convenient way for early warning [55]. This method also has a significant advantage, i.e., low cost. Given that the use of molecular biomarkers for PST monitoring mainly depends on low-cost approaches, such as PCR (Polymerase Chain Reaction) or qPCR (real-time quantitative PCR), the cost of this method is even lower than that of ELISA, which is regarded as the most cost-effective among the traditional methods of PST detection.

In this study, we screened the biomarker genes at the whole genome level, by using the following criteria: (a) the genes continuously upregulated during toxic algae exposure, and (b) showing significant correlations between gene expression and PST concentration and toxicity. Under these criteria, five genes were identified as potential biomarkers in *P. yessoensis*, among which *C1QL4-1* showed the highest correlation coefficient with both PST concentration and toxicity. Furthermore, the homolog of this gene in another species *C. farreri* also exhibited a significant correlation with both PST toxicity and concentration, implying the prevalence of this gene as a candidate biomarker to assess PST contamination in scallops. *C1QL4* is considered to be an important participant in innate immunity in invertebrates [40]. Our previous study found that some members of *C1QL4* family were involved in the responses to PST-producing algae in *C. farreri* [56]. Here, we found one of the *C1QL4* family members, *PyC1QL4-1/CfC1QL4-1,* was induced continuously during PST-producing algae exposure and its expression showed significant correlations with both PST concentration and toxicity in two scallop species, indicating that this gene might play important roles in scallops to cope with the effects of PST accumulation.

We also evaluated the practical value of the biomarker *C1QL4-1* in PST monitoring. Our results showed that *C1QL4-1* exhibited significant upregulation during the whole stage of exposure to PST-producing algae. With the increase in PST toxicity, the expression of *C1QL4-1* increased continuously. At day 1 of exposure, the PST toxicity in the whole soft-tissues of scallop was the lowest at 24.12 ± 3.59 µg STXeq/100 g (data unpublished), which was much lower than the regulatory limit of PST for commercial shellfish set by many countries (80 µg STXeq/100 g), and even lower than the tighter vigilance standards (60 µg STXeq/100 g and 40 µg STXeq/100 g). Besides, the limiting PST toxicity detected in our study on *C1QL4-1* is also much lower than the PST toxicity detected during the early stage of *A. catenella* bloom, which was reported to be reaching 75 µg STXeq/100 g [57]. Thus, *C1QL4-1* could be used as the biomarker for PST monitoring for the security and quality of farmed bivalves and early warning during the early stage of *A. catenella* bloom. In future studies, we will develop the model to predict PST toxicity in scallops with *C1QL4-1* expression to discriminate samples at the borderline concentration for commercial applications.

In addition to *PyC1QL4-1*, the other four genes also showed significant correlations between gene expression and both PST concentration and toxicity, but they could not be annotated using the currently available gene annotation information in the database. Transcriptome analysis [30] indicated only two of the four genes expressed in adult tissues of *P. yessoensis*. *Py10867.40* was specifically expressed in the digestive gland of adult *P. yessoensis*, while *Py10787.1* was highly expressed in the foot, gill, mantle, kidney, and gonad. *Py36097030.1* and *Py9125.1* were both lowly expressed (RPKM < 1) in adult tissues (Figure S1). However, the two genes expressed in adult tissues could not be found in the genome of other species, such as *C. farreri*. Thus, only the *C1QL4-1* gene is currently suitable as a biomarker for PST monitoring in scallops. In subsequent studies, we will verify its general applicability to other bivalve species.

## 5. Conclusions

In this study, the genes involved in the oxidative stress responses of scallops to PST-producing algae and a candidate biomarker for PST monitoring were identified. Transcriptome analysis showed that the genes involved in oxidation-reduction process, including *CYP*s, *IOD1*s, *PXDN*, and *ACOX1*, might be crucial in scallop digestive gland to respond to exposure to PST-producing algae. The *C1QL4-1* gene was induced continuously during PST-producing algae exposure and showed PST dosage-dependent expression in two scallop species, indicating that it may be a candidate biomarker gene for PST monitoring in scallops. Our study also demonstrated the possibility of transcriptome screening for PST biomarker identification in bivalves.

## Figures and Tables

**Table 1:** Relative toxicity of different paralytic shellfish toxins.

Toxins	Relative Toxicity
STX	1
GTX1	0.994
neoSTX	0.9243
GTX4	0.7261
GTX3	0.6379
GTX2	0.3592
C2	0.0963
GTX6	0.06
C1	0.006

## Data Availability

Data is contained within the article and Supplementary Materials.

## Supplementary Materials

The following supporting information can be downloaded at: https://www.mdpi.com/article/10.3390/antiox12061150/s1, Figure S1: The expression patterns of *Py10867.40*, *Py36097030.1*, *Py10787.1* and *Py9125.1* in adult tissues of *P. yessoensis*; Table S1: The correlation between the continuously upregulated/downregulated DEGs expression and PST concentration of the digestive gland in *P. yessoensis* exposed to *A. catenella*; Table S2: The correlation between the continuously upregulated/downregulated DEGs expression and PST toxicity of the digestive gland in *P. yessoensis* exposed to *A. catenella*.
