# Supplementary figures and images for: Transcriptome Analysis Reveals the Genes Involved in Oxidative Stress Responses of Scallop to PST-Producing Algae and a Candidate Biomarker for PST Monitoring
Source: Antioxidants (Basel). 2023 May 25;12(6):1150. doi: 10.3390/antiox12061150 (PMC10295635; doi:10.3390/antiox12061150)

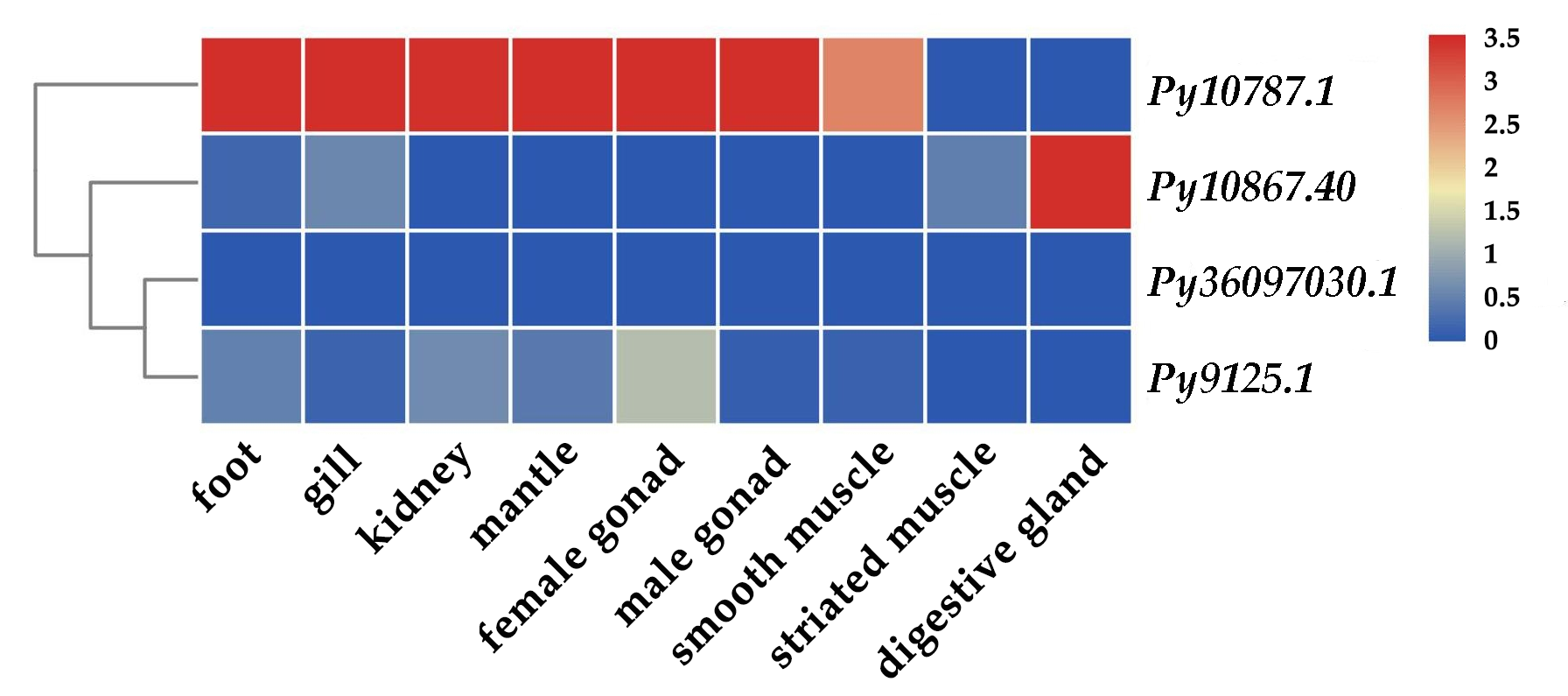

Supplement: Supplementary file 1 [file antioxidants-12-01150-s001.zip › Figure S1.tif]
